# Cuproptosis Contributes to Cisplatin-Induced Nephrotoxicity: Insights into Thymol’s Potential Inhibitory and Protective Effects

**DOI:** 10.3390/ph18111686

**Published:** 2025-11-07

**Authors:** Layla A. Al-Kharashi, Amira M. Badr, Reem T. Atawia, Elshaymaa I. Elmongy, Hanan Henidi, Rehab Ali, Awatif A. Binmughram, Lian Al-Abkka, Nervana Mostafa Kamal Bayoumy, Yasmen F. Mahran

**Affiliations:** 1 Department of Pharmacology and Toxicology, College of Pharmacy, King Saud University, P.O. Box 22452, Riyadh 11495, Saudi Arabia; lalkharashi@ksu.edu.sa; 2 Department of Pharmacology and Toxicology, Faculty of Pharmacy, Ain Shams University, Cairo 11566, Egypt; jassie_81@hotmail.com; 3 Department of Pharmaceutical Sciences, College of Pharmacy, Southwestern Oklahoma State University, Weatherford, OK 73096, USA; 4 Department of Pharmaceutical Chemistry, Faculty of Pharmacy, Helwan University, Ain Helwan, Cairo 11795, Egypt; shaymaa.taha@pharm.helwan.edu.eg; 5 Research Department, Natural and Health Sciences Research Center, Princess Nourah Bint Abdulrahman University, P.O. Box 84428, Riyadh 11671, Saudi Arabia; hahenidi@pnu.edu.sa; 6 College of Pharmacy, King Saud University, P.O. Box 22452, Riyadh 11495, Saudi Arabia; reali@ksu.edu.sa (R.A.); aalmugram@ksu.edu.sa (A.A.B.); loloalabkka@gmail.com (L.A.-A.); 7 Physiology Department, College of Medicine, King Saud University, P.O. Box 22452, Riyadh 11495, Saudi Arabia; nbayoumy@ksu.edu.sa

**Keywords:** acute nephrotoxicity, cisplatin, thymol, cuproptosis, DLAT, FDX1

## Abstract

**Background:** Cisplatin is a powerful treatment for cancer; however, its clinical application is compromised due to its potential for nephrotoxicity. The development of nephroprotective agents is hindered mainly due to the lack of understanding of the exact underlying mechanism. Additionally, the identification of safe nephroprotective agents that can be used as an adjunct to cisplatin is necessary. **Methods:** Rats were pretreated with thymol (60 mg/kg, orally) daily for two weeks and received a single cisplatin injection (8 mg/kg, i.p.) on the seventh day to induce nephrotoxicity. **Results:** Thymol prevented cisplatin-induced renal injury and restored serum creatinine and blood urea nitrogen. The renoprotective activity of thymol was further validated by histopathological studies, as demonstrated by the preserved architectures of the glomeruli, proximal, and distal convoluted tubules. Oxidative stress plays an important role in the pathophysiology of nephrotoxicity. Herein, cisplatin administration increased lipid peroxides and depleted the cellular antioxidant defense mechanisms (GSH, SOD, Nrf2, and HO-1). Interestingly, thymol remarkably ameliorated these alterations and restored oxidative status. We further examined the impact of cisplatin and/or thymol on cuproptosis, a distinct type of cell death associated with the excess intracellular accumulation of copper which is aggravated by oxidative stress. Pretreatment with thymol blunted the cisplatin-induced upregulation of genes associated with cuproptosis, including SLC31A1, DLAT, FDX1, LIAS, and ATP7A, as well as FDX1 protein expression. Furthermore, the molecular docking studies of thymol demonstrated favorable fitting and interactions with the conservative amino acids of FDX-1, DLAT, and ATP7A. This further supports the inhibitory effect of thymol on cuproptosis, which underlies its protective properties. **Conclusions:** This study illustrates that cuproptosis and oxidative stress play crucial roles in the development and progression of cisplatin-induced nephrotoxicity, and the protective activity of thymol is attributed, at least in part, to blunting these mechanisms.

## 1. Introduction

Around 20% of cancer patients are receive chemotherapy either as a standalone therapy or alongside other therapeutic modalities [1]. Since 1978, cisplatin has been successfully used to treat several types of tumors, mainly via the inhibition of DNA replication and the induction of cell death [2,3]. Kidneys accumulate cisplatin more than any other organ, and they are the primary route for excretion [4].

Unfortunately, 28–36% of patients reported acute kidney injury following an initial dose of cisplatin (50–100 mg/m^2^), which is manifested as a deterioration in kidney function and tubular damage [5,6]. As a consequence of these complications, the clinical use of cisplatin is limited and with the progression of renal injury, the intermission of cisplatin treatment remains the only option, thus compromising the treatment efficacy and escalating both healthcare costs and mortality rates [7,8]. Therefore, investigating strategies that mitigate kidney injury in cisplatin-treated patients is highly needed.

Cuproptosis represents a unique form of programmed cell death, which is triggered by intracellular copper accumulation [9]. Solute carrier family 31 member 1(SLC31A1) is the gene responsible for encoding the copper transport protein 1 (CTR1), which is crucial for intracellular copper transportation, while ATP7A/B regulate its exportation [10]. Ferredoxin-1 (FDX1) facilitates the reduction of Cu^2+^ to the more harmful Cu^+^, thus enhancing the lipoylation and aggregation of enzymes, such as dihydrolipoamide S-acetyltransferase (DLAT), a key regulator of mitochondrial metabolism [11]. This results in the aggregation of lipoylated mitochondrial proteins coupled with a decline in Fe-S cluster proteins, thereby triggering proteotoxic stress and mitochondrial dysfunction, eventually leading to cuproptosis [12]. Fe-S cluster proteins play vital biological roles, including genome stability, gene expression, electron transport chain activity, and energy generation [13].

Although the exact comprehensive mechanism of cuproptosis has not been fully investigated, critical genes that promote cuproptosis include ferrodoxin-1 (FDX1) as well as genes involved in the lipoic acid (LA) pathway, such as LA synthase (LIAS) and dihydrolipoamide dehydrogenase (DLD). In addition, genes involved in the pyruvate dehydrogenase complex, such as DLAT, are positive regulators of cuproptosis [14,15]. A recent study showed that cu+ supplementation aggravates cisplatin toxicity in vitro. Additionally, the copper importer SLC31A1 is involved in promoting the renal uptake of cisplatin and accelerating nephrotoxicity [16]. However, the exact effect of cisplatin on the cuproptosis pathway has not been fully elucidated.

Thymol is the primary constituent in thyme species and possesses a plethora of pharmacological activities, including antioxidant, anticancer, anti-inflammatory, and antibacterial activities [17,18,19]. Thymol demonstrates renoprotective effects in experimental models of kidney injury induced by high-fat diets, STZ, γ-irradiation, and cisplatin [20,21,22,23].

However, the precise mechanism by which thymol confers renoprotective effects remains unclear. The current study explored the involvement of the cuproptosis pathway in cisplatin-induced nephrotoxicity and whether thymol provides nephroprotective properties via the inhibition of cuproptosis.

## 2. Results

### 2.1. Effect of Thymol on Changes in Body Weight and Kidney Index

**Figure 1:**
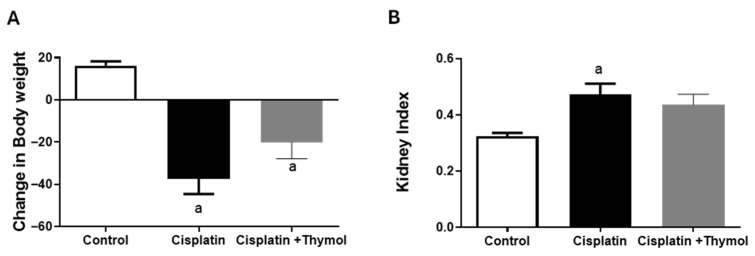
Evaluation of the effects of thymol and/or cisplatin on changes in body weight (**A**) and kidney index (**B**). Data are expressed as mean ± SEM. ^a^ *p* < 0.05 versus control group, using one-way ANOVA followed by Tukey–Kramer as the post hoc test (n = 5–7/group).

The difference in body weights on the day of sacrifice and of cisplatin injection was calculated as a change in body weight (Figure 1A), and the cisplatin-treated group showed a significant reduction in body weight compared to the control group. Thymol pretreatment tends to ameliorate the reduction in body weight, but it did not reach the statistically significant level (Figure 1A). Compared to the control group, the ratio of kidney weight to body weight significantly increased following cisplatin injection, and thymol pretreatment blunted this increase (Figure 1B).

### 2.2. Effect of Thymol on Blood Urea Nitrogen (BUN) and Serum Creatinine

**Figure 2:**
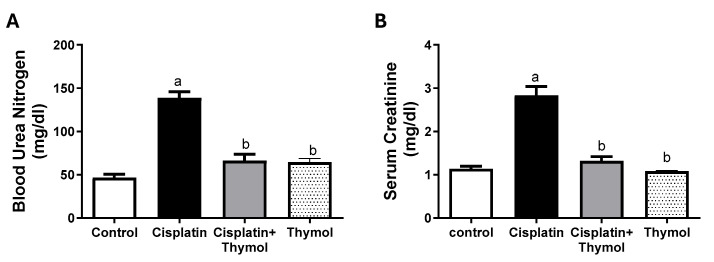
Evaluation of the effects of thymol and/or cisplatin on serum BUN (**A**) and creatinine (**B**). Data are expressed as mean ± SEM. ^a,b^: statistically different from the control group and cisplatin-exposed group, respectively, at *p* < 0.05 (n = 5–7/group).

The cisplatin-treated group showed a three-fold increase in the serum levels of BUN and creatinine compared to the control group. These alterations were restored in rats treated with thymol prior to cisplatin injection. The thymol-only-treated group showed serum levels of BUN and creatinine similar to those of the control group (Figure 2A,B).

### 2.3. Effect of Thymol on Renal Histopathology

**Figure 3:**
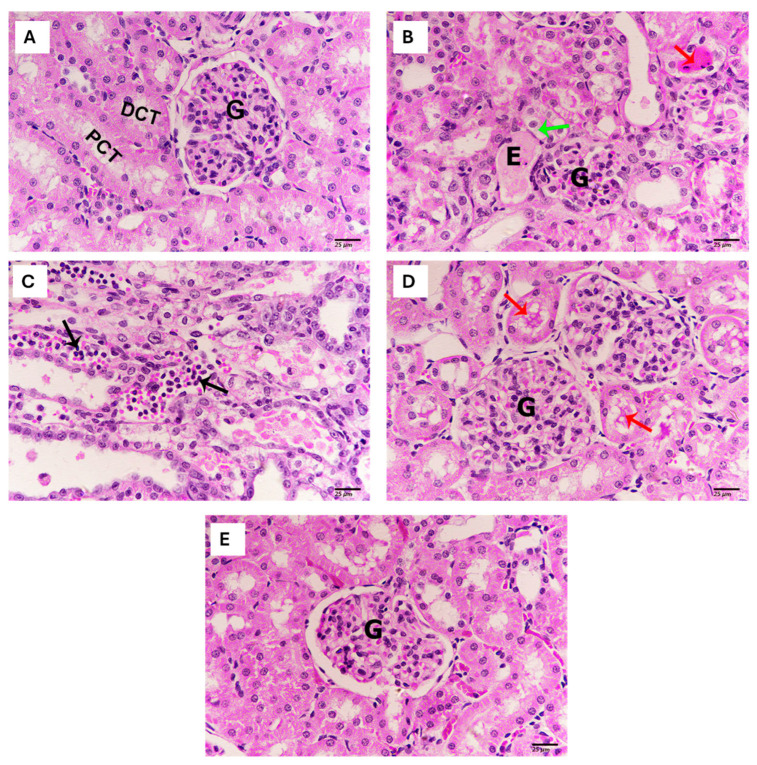
Evaluation of the effects of thymol and/or cisplatin on kidney cortex histoarchitecture. Representative photomicrograph of kidney cortex from control group (**A**) showing normal architecture, glomerulus (G), proximal convoluted tubules (PCT), and distal convoluted tubules (DCT). Sections obtained from the kidney cortex of cisplatin-treated group (**B**,**C**) showing minimized glomerulus (G), edema (E), tubular cast (black arrow), tubular degeneration (green arrow), and aggregations of inflammatory cells (red arrows). Sections of the kidney cortex from rats treated with thymol prior to cisplatin injection (**D**) revealed a healthy glomerulus (G) and tubular cast with fewer inflammatory aggregates (red arrow). (Sections obtained from the kidney cortex (**E**) treated with thymol revealing a normal structure and glomerulus (G). Scale bar = 25 µm.

The kidney cortex of the control group showed a typical architecture with abundant glomeruli and healthy tubules (Figure 3A). Meanwhile, renal tissue from rats exposed to cisplatin exhibited extensive pathological derangements such as the minimization of glomeruli, tubular degeneration, tubular casts, edema, in addition to aggregated and scattered inflammatory cells (Figure 3B,C). Moreover, renal tissue from animals treated with thymol prior to cisplatin injection revealed a marked amelioration of cisplatin-induced pathological alterations (Figure 3D). Renal tissues from animals treated with thymol revealed normal appearance, similarly to the control group (Figure 3E).

### 2.4. Effect of Thymol on Kidney Oxidative Stress

We evaluated the underlying renoprotective effects of thymol. As described in Table 1, the cisplatin-treated group demonstrated a five-fold increase in the levels of lipid peroxides, along with a significant reduction in the renal levels of reduced glutathione (GSH) and activity of the antioxidant enzyme superoxide dismutase (SOD) by 32% and 81%, respectively, as compared to the control group. No discernible differences were depicted between the thymol-only and control groups. These alterations were substantially ameliorated by the administration of thymol prior to cisplatin injection.

**Figure 4:**
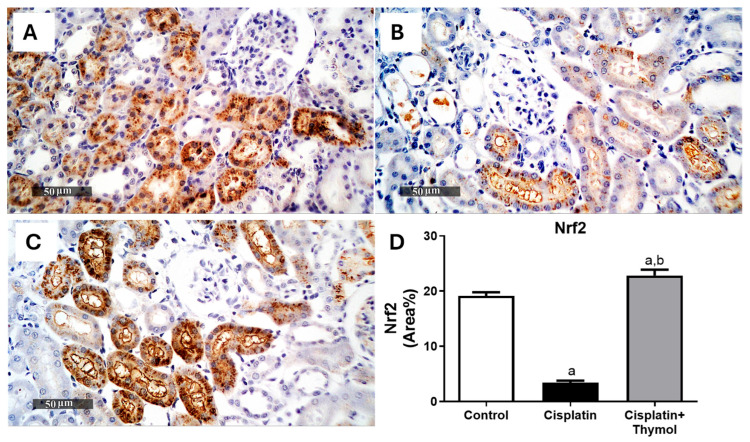
Effect of thymol and/or cisplatin on the renal expression of Nrf2. Microscopic pictures of renal Nrf2 expression in control (**A**), cisplatin-treated group (**B**), thymol+cisplatin treated group (**C**) with quantification of the % of area stained (**D**). Results are expressed as mean ± SEM (n = 3). ^a^ *p* < 0.05 versus control group and ^b^ *p* < 0.05 versus cisplatin-injected group.

**Figure 5:**
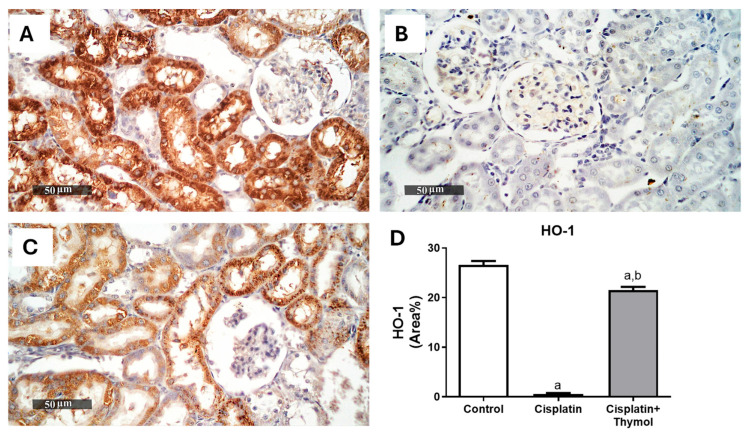
Effect of thymol and/or cisplatin on the renal expression of HO-1. Microscopic pictures of renal HO-1 expression in control (**A**), cisplatin-treated group (**B**), thymol+cisplatin treated group (**C**) with quantification of the % of area stained (**D**). Results are expressed as mean ± SEM (n = 3). ^a^ *p* < 0.05 versus control group and ^b^ *p* < 0.05 versus cisplatin-injected group.

Notably, intense immunostaining of the antioxidant regulator, nuclear erythroid-2-related factor 2 (Nrf2), and the antioxidant enzyme, heme oxygenase-1 (HO-1), were observed in sections from control rats (Figure 4A and Figure 5A). The percentage of immunostaining was dramatically reduced in the cisplatin-treated group (Figure 4B and Figure 5B). In contrast, pretreatment with thymol (60 mg/kg) significantly maintained the expression levels near the control levels (Figure 4C and Figure 5C).

### 2.5. Effect of Thymol on Markers of Cuproptosis

**Figure 6:**
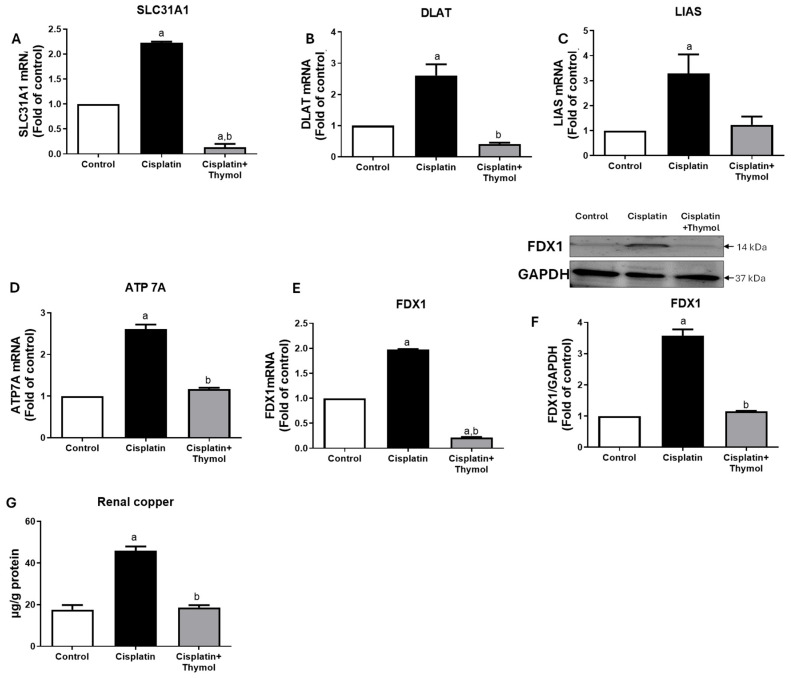
Effect of thymol and/or cisplatin on renal mRNA expression of cuproptosis-related genes. mRNA expression of SLC31A1 (**A**), DLAT (**B**), LIAS (**C**), ATP7A (**D**), and FDX1 (**E**) in the renal tissues of control rats or cisplatin-injected rats or thymol-treated rats prior to cisplatin. Protein expression of FDX1 with quantifications (**F**). Renal concentration of copper following exposure to cisplatin and/or thymol (**G**). Results are expressed as mean ± SEM (n = 3–4). ^a^ *p* < 0.05 versus control group and ^b^ *p* < 0.05 versus cisplatin-injected group, using one-way ANOVA followed by Tukey–Kramer as post hoc test. SLC31A1, solute carrier family 31 member 1; DLAT, dihydrolipoamide S-Acetyltransferase; FDX1, ferredoxin 1; LIAS, lipoic acid synthase; and ATP7A, ATPase copper-transporting alpha.

We further evaluated the influence of thymol on the expression of genes involved in cuproptosis, a copper-mediated cell-death pathway, using rt-PCR. Exposure to cisplatin induced a significant two-to-three-fold increase in solute carrier family 31 member 1 (SL31A1, Figure 6A), dihydrolipoamide S-Acetyltransferase (DLAT, Figure 6B), lipoic acid synthase (LIAS, Figure 6C), ATPase copper-transporting alpha (ATP7A, Figure 6D), and ferredoxin 1 (FDX1, Figure 6F) as compared to the control group. Interestingly, pretreatment with thymol blunted the increase in cuproptosis-related genes induced by cisplatin to near control levels (Figure 6). Moreover, FDX1 protein expression was significantly increased in the kidneys of rats exposed to thymol, while treatment with thymol significantly restored FDX1 protein levels (Figure 6F). The effect of cisplatin on cuproptosis was further supported by analyzing renal copper levels, and our data revealed increased renal copper levels following cisplatin exposure. Interestingly, pretreatment with thymol restored copper levels to near control levels (Figure 6G).

### 2.6. Molecular Docking Studies

Molecular docking was performed on three cuproptosis-related biomarkers; one was the crystal structure FDX-1 along with its co-crystallized ligand (PDB ID:3P1M), while the other was the DLAT protein which was downloaded with (PDB ID: 3B8K), and the third crystal structure was ATP7A with (PDB ID: 7LU8).

**Figure 7:**
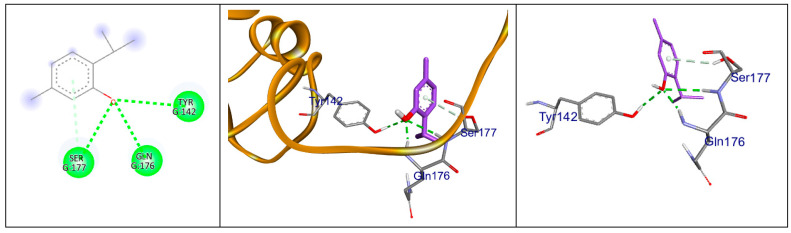
Docking of thymol (present in magenta sticks) on FDX-1 (pdb:3P1M); hydrogen bonds are in the green dashed lines and pi-donor hydrogen bond in light green dashed lines.

**Figure 8:**
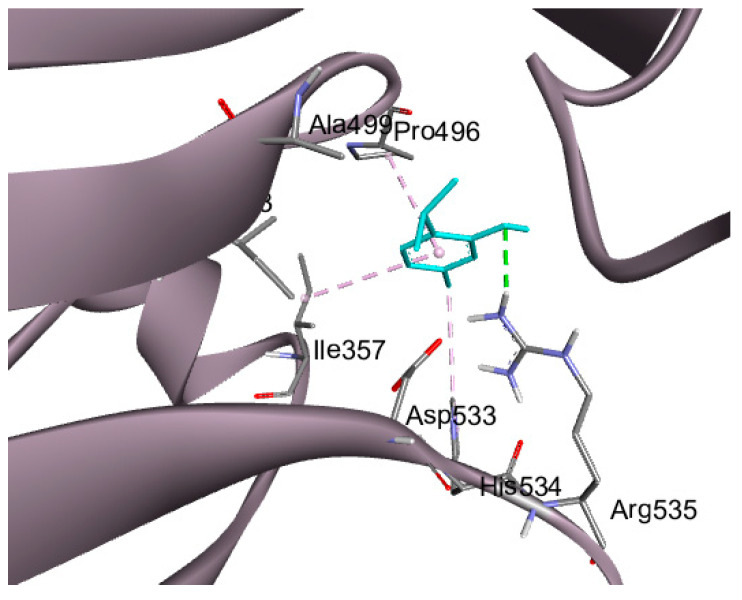
Docking of thymol (presented in blue sticks) on DLAT (Pdb: 3b8k); hydrogen bonds are in the green dashed lines and hydrophobic interactions are in the violet dashed lines.

A recent study revealed a distinctive type of copper-dependent cell death known as cuproptosis, which is closely associated with ferredoxin 1 (FDX1), a protein involved in the regulation of lipoylation [14,24]. Thymol showed a significant interaction with the downloaded crystalized protein with three hydrogen bonds with Ser 177, Gln 176, and Tyr 142, in addition to a fourth non-conventional pi-donor hydrogen bond with Ser 177 residue, as illustrated in Figure 7. Cuproptosis is a form of cell death brought on by an accumulation of copper in the mitochondria, which causes the protein lipoylated dihydrolipoamide S-acetyltransferase (DLAT), which is connected to the mitochondrial tricarboxylic acid (TCA) cycle, to aggregate. Proteotoxic stress is the end outcome of this process, which causes cell death [25]. Thymol interaction with DLAT with binding energy −4 kcal/mol formed one hydrogen bond and three hydrophobic interactions, as shown in Figure 8.

**Figure 9:**
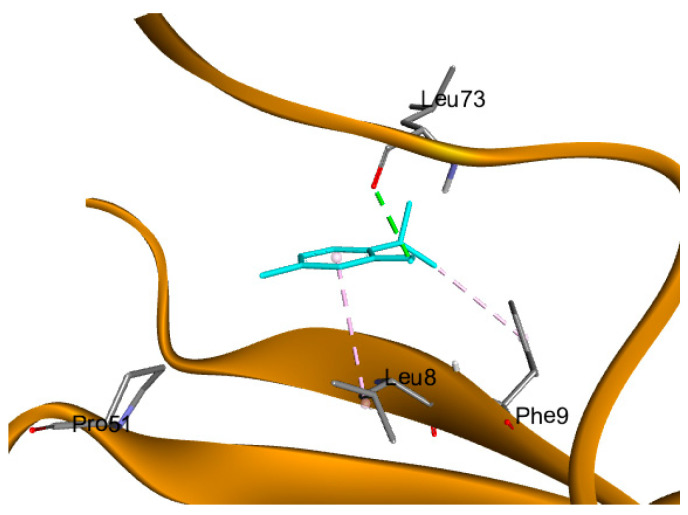
Docking of thymol (present in blue sticks) on ATP7A (pdb: 7LU8), where hydrogen bonds are in the green dashed lines and hydrophobic interactions are in the violet dashed lines.

ATP7A inhibitors are compounds that target and block the ATP7A enzyme’s activity, which modifies the body’s transport of copper. The ATP7A enzyme may become bound by these inhibitors, changing its structure and impairing its ability to perform transport tasks. On the other hand, they can obstruct the enzyme’s capacity to hydrolyze ATP, a process that is essential for the transport of copper. These inhibitors can impact a number of downstream processes that rely on the distribution and availability of copper by interfering with ATP7A action. Accordingly, ATP7A was selected for the docking of thymol, aiming to support the biological investigations. Thymol demonstrated a hydrogen bond interaction with LEU 73 amino acid residue in addition to two hydrophobic interactions, as illustrated in Figure 9. The binding energy of docking results in addition to the type of interactions with the amino acid residues are tabulated in Table 2.

### 2.7. PCA and Hierarchical Clustering Heatmap

**Figure 10:**
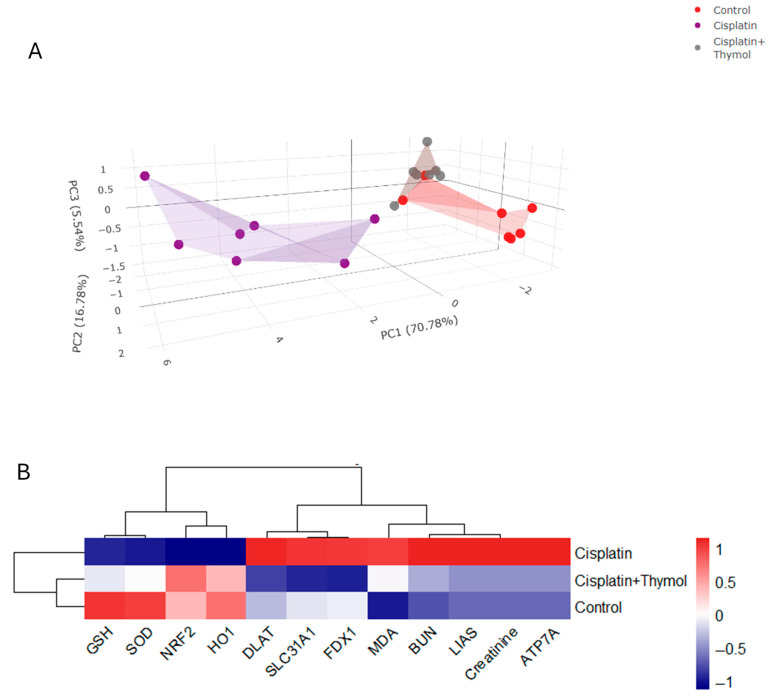
Multivariate analyses of cisplatin and/or thymol treatment. 3D score plot of PCA for classifying the three experimental groups (control, cisplatin, and cisplatin+thymol). PC1 (71.78%), PC2 (16.78%), and PC3 (5.54%) are shown on the axes (**A**). Heatmap cluster showing variation in assessed data sets among the different treatment groups (**B**).

The multivariate analysis among the different treatment groups was assessed using PCA. Variables were classified into three principal coordinate components (PC1, PC2, and PC3). The majority of the analyzed variables were differentiated by PC1, which accounts for the highest proportion of variance (70.78%), followed by PC2 (16.78%) and PC3 (5.54%). PC1, PC2, and PC3 collectively account for 93.1% of the variance. As seen in Figure 10A, the PCA revealed that the control and cisplatin+thymol groups were clustered together and isolated from cisplatin, particularly along the PC1. The clustering heatmap (Figure 10B) provides a visual representation of BUN, creatinine, GSH, MDA, SOD, NRF2, HO1, ATP7A, LIAS, FDX1, SLC31A1, and DLAT which demonstrates the significant variation between the levels of analyzed variables among the different treatment groups. These data show the substantial distinction of the cisplatin-treated group.

## 3. Discussion

Despite the high efficacy of cisplatin as an anti-neoplastic agent for multiple solid tumors, its widespread use is limited by its nephrotoxic effects in 25–35% of patients following a single injection [26]. This is mainly attributed to its selective accumulation in the renal cortex [27,28]. Despite extensive studies on unraveling the cellular and molecular mechanisms of cisplatin-induced nephrotoxicity, restoring kidney function remains a difficult challenge. Several experimental studies demonstrated the contribution of oxidative stress, inflammation, cell death, diminished renal blood flow, and epigenetic mechanisms [29]. A critical barrier to reducing the prevalence of cisplatin-induced nephrotoxicity is understanding its pathogenesis and underlying molecular mechanisms.

In this study, we demonstrated that a single dose of cisplatin induced an accumulation of BUN and serum creatinine, two reliable markers of kidney function. This indicates the reduced capacity of the kidney to filter and eliminate nitrogenous byproducts. Previous studies showed that patients with increased serum creatinine following cisplatin injection suffer acute renal failure [30]. Interestingly, the administration of thymol ameliorated BUN and serum creatinine to control levels as well as improved the histopathologic alterations induced by cisplatin, which highlights the nephroprotective properties of thymol. These results are in line with the previous data [21,23,31].

Notably, the nephroprotective effects of thymol were not associated with the amelioration in body weight reduction induced by cisplatin. This suggests that the effects of thymol on renal tissues are mainly due to local effects on kidney. Thus, we further evaluated the mechanisms underlying the nephroprotective effects of thymol. Herein, we demonstrated that thymol effectively blunted the oxidative stress associated with cisplatin.

Previous studies showed that a cisplatin-induced increase in reactive oxygen species is the main contributor to kidney injury. Increased ROS is implicated in the damage of cellular components such as DNA, lipids, and proteins as well as cellular organelles suchas mitochondria and the endoplasmic reticulum [32,33,34]. An important regulator of the cellular redox homeostasis is the antioxidant transcription factor, Nrf2, which activates antioxidant-reactive elements (AREs) to regulate the expression of several antioxidant genes including the gene encoding for HO-1 [35,36]. HO-1 is an important mediator of the antioxidant effects of Nrf2. HO-1 induces the catabolism of free heme into iron, carbon monoxide, and bilirubin, which are powerful antioxidants and play an important role in scavenging ROS, thus alleviating oxidative stress [37]. Several lines of evidence showed that cisplatin downregulated Nrf2/HO-1 levels [38,39], which corroborates with our study suggesting that targeting Nrf2/HO-1 pathways can alleviate cisplatin-induced nephrotoxicity. Additionally, the antioxidant properties of thymol have been previously demonstrated through the upregulation of Nrf2/HO-1 expression [40,41], increased SOD and GSH levels, as well as reduced lipoperoxidation [42]. Our study supports that these favorable effects of thymol are evident in the context of cisplatin-induced nephrotoxicity.

Cuproptosis is a process of tightly regulated cell death which involves the accumulation of copper (Cu) [14]. Under normal conditions, the maintenance of Cu homeostasis is essential for vital biological processes [43]. However, the disruption of Cu homeostasis induces oxidative stress and cell death. Increased intracellular accumulation of copper results in the oligomerization and aggregation of mitochondrial lipoylated proteins, such as DLAT, and the loss of Fe-S cluster proteins, which in turn induces proteotoxic cellular stress and cell death. Additionally, FDX1 is involved in the reduction of Cu^2+^ to the more toxic Cu^+^, which accelerates protein aggregation and cellular stress [14].

Previous studies have shown that ROS accumulation induces cuproptosis [14,44]. Interestingly, the deletion of NRF2 has been associated with increased expression of the cuproptosis-related genes FDX1, DLAT, and lipoyl synthase (LIAS) [45]. Also, the depletion of GSH, which acts as a copper chelator, is associated with enhanced cuproptosis [46]. Cuproptosis has been involved in the progression of several cardiovascular and neurocognitive disorders, as well as liver and renal injury [47,48,49,50]. Additionally, our results align with a previous study that highlights the protective effect of a cuproptosis inhibitor (ammonium tetrathiomolybdate) against cisplatin-induced acute kidney injury, mainly via the activation of the NRF2 signaling pathway and relieving oxidative stress [51]. The cross-talk between ROS generation/oxidative stress and cuproptosis has been described in [52]. Also, the mechanism of ROS-induced cuproptosis potentially involves increased copper influx via the upregulation of cellular copper importers such as SLC31A1, thereby upregulating FDX1 in the presence of copper [53].

Intracellular copper concentration is regulated by the copper importer SLC31A1 (CTR1) and the copper exporters ATP7A and ATP7B [54,55]. A recent study demonstrated the role of cisplatin-induced upregulation of SLC31A1 in the progression of nephrotoxicity, while SLC31A1 silencing counteracts cisplatin-induced cell death and nephrotoxicity [16]. This is in accordance with results from our study; however, our data expands our knowledge on the effect of cisplatin on cuproptosis-related genes and the assessment of renal copper levels. Interestingly, thymol blunted cisplatin-induced expression of cuproptosis-related genes and renal copper levels. Although several studies showed that the expression of the copper exporter ATP7A negatively correlates with cuproptosis [56,57], our data showed that cisplatin induced the expression of ATp7A, which could be explained as a compensatory mechanism of renal cells in response to increased copper accumulation. Also, to our knowledge, this is the first study to investigate the potential impact of thymol on cuproptosis via increased Nrf2/HO-1 and GSH levels.

Moreover, docking results showed potential binding affinities between thymol and the amino acid residues of FDX1, DLAT, and ATP7A binding pockets. This further suggests a direct interaction resulting in the negative regulation of the cuproptosis pathway. The multivariate analyses using PCA further supported the notable distinction of the cisplatin group from other treatment groups, mainly along the PC1 axis (71.78%). Additionally, the heatmap visualization of the assessed data sets shows a clear discrimination of the cisplatin-treated group from other groups in the assessed data sets. Thus, the current study provides in silico and in vivo for the protective effects of thymol against cisplatin-induced nephrotoxicity, potentially via the attenuation of the cuproptosis pathway and oxidative stress.

## 4. Materials and Methods

### 4.1. Drugs and Chemicals

Cisplatin was obtained from Hospira, UK, Ltd. (Royal Leamington Spa, UK). Thymol (2-isopropyl-5-methylphenol) was purchased from Abcam, located in Cambridge, UK. The highest commercially available grade was utilized for all other chemicals.

### 4.2. Animals

Male Wistar rats, aged approximately 10 weeks and weighing 150–200 g, were acquired from the animal facility at the Faculty of Pharmacy, King Saud University, Riyadh, Saudi Arabia. Rats were housed under standard conditions at a temperature of 25 °C, with a 12 h cycle of light and darkness and had unlimited access to a standard diet and water. In accordance with the ethical guidelines of the King Saud Scientific Research Ethics Committee (IRB number: KSU-SE-20-52, approved on 15 September 2024) at the Faculty of Pharmacy, King Saud University, all animals were acclimated for one week prior to the start of the experiment.

### 4.3. Experimental Design and Sample Preparation

The rats were randomly allocated to four groups of eight animals each. Group 1 serves as the control group and received daily oral administration of a vehicle consisting of 0.5% Tween in normal saline daily for two weeks and a single injection of saline on Day 7 (i.p.). Group 2, designated as the disease group, was administered the vehicle orally for two weeks and received a single cisplatin injection (8 mg/kg, i.p.) on Day 7 to induce nephrotoxicity. The treated group, designated as Group 3, was given thymol (60 mg/kg, orally) daily for 2 weeks and received a single cisplatin injection (8 mg/kg, i.p.) on Day 7. Group 4, serving as the drug-only treated group, was administered thymol orally daily for two weeks. Doses for cisplatin and thymol were determined based on prior studies from our lab [20,22]. All animals were weighed on the first and seventh days of the experiment to determine the dose. Upon completion of the study, the rats were weighed before being euthanized. Trunk blood was collected from the different treatment groups and centrifuged at 1000× *g*. Then, sera were divided into aliquots and stored at −80 °C. Kidneys were excised, and their weights were recorded. Portions of the kidneys from different treatment groups were preserved in 10% formaldehyde for histopathological evaluation. A different section of the kidney was rapidly frozen using liquid nitrogen for the polymerase chain reaction (PCR) analyses, or homogenized in ice-cold phosphate-buffered saline, and supernatants were collected for the assessment of oxidative stress markers and protein levels biochemically.

### 4.4. Evaluation of Markers of Kidney Function

Serum creatinine and blood urea nitrogen (BUN) were determined for the different treatment groups using the commercially available kits (United Diagnostics Industry, Dammam, Saudi Arabia) following the provided instructions. The kidney index was determined by dividing kidney weight (mg) by body weight (g).

### 4.5. Evaluation of Renal Histopathological Architecture

Renal tissues were preserved for 24 h in 10% formalin (pH 7.4), followed by dehydration and paraffin embedding. Renal sections were sliced and stained with hematoxylin and eosin (H&E). Slides from the different treatment groups were examined using light microscopy to detect pathological alterations in glomeruli and tubules.

### 4.6. Evaluation of Renal Nrf2 and HO-1

Renal expressions of Nrf2 and HO-1 were evaluated using the immunohistochemistry (IHC) technique as previously described [22], using the primary antibody against rat NRF2 or HO1(1:100, Thermo Fisher Inc., Waltham, MA, USA), then incubated with the corresponding secondary antibody and counterstained with hematoxylin for examination. The percentage of positively stained areas were analyzed in six randomly selected independent fields using a Leica Application module associated with the imaging system.

### 4.7. Evaluation of Markers of Oxidative Stress and Protein Levels

The commercially available assay kits from (Biodiagnostic Co., Giza, Egypt) were used to measure the activity of the antioxidant enzyme, superoxide dismutase (SOD), levels of reduced glutathione (GSH), and lipid peroxides, assessed as thiobarbituric acid-reactive substances (TBARSs) in kidney homogenates [58]. Protein levels were assessed using the Bradford method [59].

### 4.8. Evaluation of Renal mRNA Expression of Cuproptosis Markers (SLC31A1, DLAT, FDX1, LIAS, and ATP 7A)

Total RNA was extracted from renal tissues using a PureLink RNA Micro Kit (Invitrogen, Waltham, MA, USA) following the manufacturer’s protocol. cDNA was generated via reverse transcription using a commercial kit (Thermo Scientific, Waltham, MA, USA). Real-time PCR analysis was conducted using the SYBR green master mix, rat-specific primers (Table 3) and a light-Cycler instrument V.2 (Roche, Indianapolis, IN, USA). Gene expression was normalized to β-actin, computed using the (2^−ΔΔCt^) formula, and expressed as fold change of the control.

### 4.9. Protein Expression of FDX1

The protein expression of FDX1 was analyzed using Western blotting as previously described [42]. Briefly, proteins were isolated, separated on the SDS-PAGE and transferred to PVDF membranes (Bio-Rad, Hercules, CA, USA) and the housekeeping glyceraldehyde-3-phosphate dehydrogenase (GAPDH, Cat.# ab8245). Protein expressions were quantified densitometrically using the ImageJ software1.54 (NIH Image, Bethesda, MD, USA).

### 4.10. Assessment of Renal Copper Levels

Renal concentrations of Copper (Cu^2+^) were determined colorimetrically at 580 nm using a complexing assay kit (Cat.#E-BC-K300-M, Elabscience, Wuhan, China) according to the manufacturer’s instructions.

### 4.11. Docking Studies

Molecular modeling studies were conducted, for which proteins were obtained from the Protein Data Bank for docking purposes. The structure of thymol was first drawn as a 2D diagram using ChemSketch (https://www.acdlabs.com/resources/free-chemistry-software-apps/chemsketch-freeware/, accessed on 2 November 2025), then converted to 3D with Open Babel and saved in SDF format, compatible with UCSF Chimera 1.17.3 and Discovery Studio. The ligand and protein were prepared in UCSF Chimera by adding hydrogen atoms and assigning Gasteiger charges, followed by energy minimization. A grid box was created using AutoGrid, centered on the macromolecule, and then resized to encompass the entire protein. Docking was executed with AutoDock Vina, and the results were visualized with Biovia Discovery Studio v21.1.0.20298 [60,61].

### 4.12. Statistics

The GraphPad Prism software version 10 (GraphPad, San Diego, CA, USA) was used for statistical analyses and graph presentation. Statistical difference among multiple treatments was calculated using one-way ANOVA and Tukey–Kramer post hoc tests. A *p*-value of less than 0.05 was considered statistically significant. Data are expressed as means ± SEM. The principal component analysis (PCA) and hierarchical clustering heatmap were generated using R Studio (R version 4.4.1).

## 5. Conclusions

Collectively, this study provides evidence for the protective mechanisms of thymol against cisplatin-induced renal injury. The underlying molecular mechanism mainly involves blunting the expression of cuproptosis-related genes including SLC31A1, FDX1, DLAT, LIAS, and ATP7A, induced by cisplatin injection. Additionally, this study sheds light on the interplay between the antioxidant effects of thymol via the activation of the Nrf2/HO-1 pathway and the negative effects on cuproptosis. These findings pave the way for further investigations of thymol as a natural modulator of the cuproptosis pathway and its role in ameliorating cisplatin therapy-associated nephrotoxicity. Moreover, a more detailed understanding of the role of cuproptosis in cisplatin-induced toxicities may help in the management and improvement of the therapeutic outcomes.

## Figures and Tables

**Table 1:** Effects of thymol (60 mg/kg) and/or cisplatin on lipid peroxides, GSH, and SOD.

Group	MDA (nmol/mg Protein)	GSH (µg/mg Protein)	SOD (U/mg Protein)
Control	0.54 ± 0.05	1.83 ± 0.03	4.46 ± 0.1
Cisplatin	2.45 ± 0.07 ^a^	0.59 ± 0.04 ^a^	0.83 ± 0.1 ^a^
Cisplatin+Thymol	1.46 ± 0.08 ^a,b^	1.12 ± 0.03 ^a,b^	2.61 ± 0.043 ^a,b^
Thymol	0.52 ± 0.06 ^b,c^	1.7 ± 0.1 ^b,c^	4.78 ± 0.29 ^b,c^

Data are represented as means ± SE (n = 5). ^a,b,c^: Significantly different from the control group, cisplatin group, and thymol+cisplatin group, respectively, at *p* < 0.05. MDA, malondialdehyde; GSH, glutathione; and SOD, superoxide dismutase.

**Table 2:** Binding energy and type of interactions between thymol and the amino acid residues of target proteins.

Protein	PDB ID	Binding Energy Kcal/mol	RMSD (Å)	Amino Acid Residues of Interaction	Types of Bonds
FDX-1	3P1M	−3.81	1.67	Ser 177	H-bond
				Ser 177	Pi-donor H-bond
				Gln 176	H-bond
				Tyr 142	H-bond
DLAT	3B8K	−4.5	2.0	ARG 535	H-bond
				ILE 357	Pi-alkyl hydrophobic
				HIS 534	Pi-alkyl hydrophobic
				Pro 496	Pi-alkyl hydrophobic
ATP7A	7LU8	−3.86	1.21	Leu 73	H-bond
				Leu 8	Pi-alkyl hydrophobic
				Phe 9	Pi-alkyl hydrophobic

FDX-1; ferredoxin 1, DLAT; dihydrolipoamide S-acetyltransferase, ATP7A; ATPase copper-transporting alpha, and RMSD; root mean square deviation.

**Table 3:** Primer sequences used in real-time PCR.

Genes	Primer	Sequence (5′-3′)	Accession Number
*DLAT*	Forward	TGGACCCCGGCTCTTCTCTT	NM_031025.1
	Reverse	CATTCCAGGGCTTCTCCACT:	
*FDX1*	Forward	CCAGCGTGGAGCGAGTTTG	NM_017126.2
	Reverse	TGGCTCCAGGGTTTGTTGTC	
*LIAS*	Forward	TCCACTCCTGATCTTGGACAC	NM_001012037.1
	Reverse	TGGTGCTTCTTGTGGAGTAA	
*SLC31A1*	Forward	GGGCTTGGGAGAAGTCCAGA	NM_133600.3
	Reverse	TCCTCATGTGGTCCGAAGGA	
*ATP7A*	Forward	CCAGCGTGGAGCGGACTAC	XM_039099486.2
	Reverse	TGGCTCCAGGGTGTCATCTT	
*β-actin*	Forward	CCTGCTTGCTGATCCACA	NM_031144.3
	Reverse	CTGACCGAGCGTGGCTAG	

## Data Availability

The original contributions presented in this study are included in the article. Further inquiries can be directed to the corresponding author.
